# Constructing populations in biobanking

**DOI:** 10.1186/s40504-015-0024-0

**Published:** 2015-07-21

**Authors:** Aaro Tupasela, Karoliina Snell, Jose A. Cañada

**Affiliations:** Department of Public Health, Centre for Medical Science and Technology Studies, Øster Farimagsgade 5, PO Box 2099, 1014 Copenhagen, Denmark

**Keywords:** Biobanking, Bio-objects, Engagement, Governance, Populations, Standards

## Abstract

This article poses the question of whether biobanking practices and standards are giving rise to the construction of populations from which various biobanking initiatives increasingly draw on for legitimacy? We argue that although recent biobanking policies encourage various forms of engagement with publics to ensure legitimacy, different biobanks conceptualize their engagement strategies very differently. We suggest that biobanks undertake a broad range of different strategies with regard to engagement. We argue that these different approaches to engagement strategies are contributing to the construction of populations, whereby specific nationalities, communities, societies, patient groups and political systems become imbued or bio-objectified with particular characteristics, such as compliant, distant, positive, commercialized or authoritarian. This bio-objectification process is problematic in relation to policy aspirations ascribed to biobanking engagement since it gives rise to reified notions of different populations.

## Introduction

The success of any research project has a direct relation with the trust of participants that will voluntarily accept to provide samples and, usually, private information about their health and living habits. To secure the trust of all partners, including participants and the larger community to which they belong, public information, consultation and public participation seems one of the most important factors influencing success. (European Union 2010, 131)

Recently, Kowal (2013) has noted that no research using samples and information is safe, unless researchers establish and maintain relationships with the communities from which samples come from. This normative observation may provide important insight into the development and maintenance of sustainable global biobanking initiatives and their relationship with the local/regional/national/global context in which they are embedded. Public participation and involvement is by no means a new topic in relation to techno-political legitimacy. Arnstein’s (1969) ladder of participation is a classic example of the ways in which involvement has been made a political issue in relation to planning and development where increased levels of involvement are seen to be desirable.

This article poses the very simple question of whether biobanking practices and standards related to the ethical, legal and social aspects of biobanking are, in fact, giving rise to, and construction of populations from which biobanking initiatives (national, regional, local, disease-based, patient group based etc.), such as deCode, 23andMe, CARTaGENE and UK Biobank, draw on for legitimacy? Recent biobanking policies encourage various forms of engagement with populations to ensure legitimacy (European Union 2010), yet different countries and biobanks conceptualize their engagement strategies very differently. Snell et al. (Snell, Karoliina, Jose A. Cañada, and Aaro Tupasela: Strategies and practices of biobank engagement, submitted.) have develop a typology of public engagement from the viewpoint of the biobanks where they suggest that although there are a number of policy guidelines for engagement, biobanks in fact, undertake a broad range of different strategies with regard to engagement. We argue in this paper that these different approaches to engagement strategies are also giving rise to the construction of populations, whereby specific nationalities, populations, patient groups and political systems become imbued with particular characteristics, such as deliberative, consensus seeking, commercialized or authoritarian (cf. Jones and Salter 2003). We see this as a type of co-construction of identity, whereby the population from which the biobank draws from, helps to define and characterize the biobank, but also that the identification, collection and distribution of samples and data also give rise to the construction of a population at the same time (cf. Appiah 2005). This co–construction can also be understood as a form of bio-objectification whereby the governance of biobanks give rise to popular conceptualizations of the population they draw their material from (Vermeulen et al. 2012). The processes of bio-objectification of populations, however, are not uniform, but rather take on different characteristics and paths depending on the types of engagement practices that various biobanks undertake in their operations. In addition, we see the process of bio-objectification as an iterative process between historical, political and scientific activities where these different spheres interact with each other in different configurations and ways. The process of bio-objectification gives rise to forms of legitimation through which local, regional, national and supra—national actors seek to leverage and utilize samples in a more efficient way. We see this process contributing to the politicization of populations in Europe and elsewhere, whereby groups of people are ascribed particular types of characteristics (not only in a medical sense, but also in a public opinion and political action meaning as well) in relation to engagement and biobank perspectives, as opposed to understanding public opinion and perceptions as being fluid and changing over time, requiring regular interaction and dialogue (Tupasela Aaro: Branding populations in medical research – placing genes on the global genetic atlas, submitted).

These construction processes draw on a broad range of influences and activities related to the ethical, legal and social aspects of managing biobanks. The influences may be drawn from historical, cultural, political, scientific spheres or any other source, which can be leveraged to construct populations, which are seen to be more compliant and supportive of biobanking in general. In a general sense, the process can be seen as an attempt at stabilizing the populations from which biobanks draw their material from (cf. Douglas 2012). One important example of engagement has often been seen the production of population surveys, ethnographic studies of populations (Kowal 2013; Pálsson 2007), case studies of specific biobanks (Haimes and Whong-Barr 2003), focus group studies (Snell et al. 2012), ELSI studies on biobanking (Kaye et al. 2012; Hardcastle 2007) or other studies, which have sought to explore the underpinning of human tissue procurement and use (Rabinow 1999). These perspectives, although providing important insights into the local and regional perspectives of people regarding biobanking also have an unintended consequence of standardizing conceptions of attitudes, which can be used in many cases to draw on for legitimacy. We see this as contributing to the bio-objectification of populations, which can also be understood in some respects as a form of population branding (Tupasela 2012). These studies have also contributed to constructions of racialized notions of populations (Reardon and TallBear 2012; TallBear 2003). According to Igo (2007), this contributes to the development of a notion of self, giving rise to certain types of vocabulary that are affiliated with particular populations or groups of people. Some have also suggested that this can also give rise to false notions of consensus regarding specific issues, such as informed consent (Master et al. 2012). Besides biobanks drawing on reified notions of common history and national identity (Schwartz-Marin and Silva-Zolezzi 2010), they can also be seen as contributing to normative assumptions on the opinions of the people who participate in them, or more specifically the people who are surveyed and interviewed for their opinions on them.

One of the most common motifs of vocabulary draws from that of participatory engagement. Participatory models of engagement rely to a certain extent on an understanding that increased participation also increases the flow of information in both directions, as opposed to being top-down in nature. From a governance perspective, such a position may hold important possibilities for interaction in relation to biobanking. Some have argued that increased involvement makes visible the ‘patient work’ (Corbin and Strauss 1985) and ‘clinical labor’ (Cooper and Waldby 2014) that sample donors provide in order for collections to become possible and operable, as well as allows for increased responsiveness to the interests and concerns of those who have participated. Community engagement is seen as a central aspect of any biobanking governance scheme (Halderman et al. 2014; O’Doherty et al. 2011; Shalowitz et al. 2009). The basic theoretical assumptions underlying engagement with the research population stem from an understanding and belief that engaging with the research population will bring with it improved understanding of the context in which research takes place, as well as improved results in the outcomes of the research and public health (NIH 2011).

Studies of patient activism are another example, which have indicated that patient organizations are increasingly involved in the organization of research and sample acquisition surrounding specific diseases and rare conditions (Rabeharisoa and Callon 2002). These novel knowledge-making coalitions between patients and research organizations, as well as companies, highlight the ways in which citizen and patient participation is in some cases becoming more prevalent and inclusive in biomedical research (Epstein 2007), but also the ways in which patient organizations are increasingly becoming a political apparatus for mobilization of resources. This mobilization and the legitimacy that it carries are in part based on standardized notions of patient organizations and their members as actively interested in such forms of participation. Callon and Rabeharisoa (2008) note that such groups play an increasingly important role in the development of technoscience, politics, and economic life. Yet in many cases patient organizations are not as active in engaging with biomedical research, but rather far more involved in providing peer support to their members. If indeed biobanking and the supporting infrastructure surrounding the systems through which information on patients will be collected, stored and studied, the concern with the forms and styles of engagement and governance become all the more significant.

We argue, however, that the certain specificities within national, regional and institutional contexts are giving rise to the construction and bio-objectification of populations and publics in relation to biobanking, whereby engagement is understood in a very diverse manner that rises in connection with the individual context of a given biobank. The way public engagement is constructed and understood by different biobanks in their individual contexts is from where arguments that validate their legitimacy draw from. In our analysis we are drawing on the notion of populations as a collection of individuals which are studied and acted upon scientifically and medically. The notion of public, however, is used more to connote a political body of people which is engaged with. These two conceptions, however, are not separable in that in biobanking the populations which are studied and medically acted upon are also necessarily publics which have political significance. What is of interest, however, is the ways in which the two categories become collapsed and positioned differently depending on each biobanks engagement strategy.

The empirical material analyzed in this research is based on a two-year project, which examined and explored the forms and styles of engagement that various biobanks undertook in six countries: USA, Canada, UK, Spain, Finland and Iceland. The scope of our research focused on engagement strategies of biobanks in these different countries. In our research we conducted 16 semi-structured interviews with relevant personnel from different biobanks, as well as biobank networks in the six countries. We also conducted six interviews with policy makers and regulators to develop a broader picture of the nature of engagement undertaken by various biobanks in the different countries. Policy makers were defined as people who worked solely in government ministries or regulatory bodies, since in many cases it became apparent that biobank managers also had a significant impact on the ways in which national biobank policies were developed. Furthermore, in many countries the regulatory process of rewriting legislation and policy often involves an interative process between various stakeholders, which blurs the categorical differentiation between various actors. We supplemented the interviews with international (European Commission 2012; OECD 2009) and national policy documents from the various countries (National Cancer Institute 2011), as well as different types of documents that we could find that had been published by or about the various biobanks, which we were studying (Auria Biobank 2014). These documents included different types of white papers and preparatory documents, as well as national legislations and recommendations (cf. Biobank Act 2012). In selecting the countries and biobanks that we would study, we sought to cover a broad range of different types of biobanks, as well as regions, taking into account both the size of the country, type of healthcare systems they had, as well as the legal structures which are in place. For this article, we selected Canada, Spain and Finland since they are very different in terms of their approaches to biobanking and historically diverse. We draw examples from these countries in order to show the ways in which populations are becoming increasingly constructed as a way through which legitimacy is drawn from.

In the following, we will briefly discuss the issue of constructed population and the normative problems it has in relation to the legitimation of biobanking activities. We will then draw on our empirical material to provide some concrete examples of the ways in which this has become manifested in different contexts.

### Constructing biobank populations

A number of recent studies have suggested that populations have become associated with varying conceptions of race, ancestry and ethnicity, which derive legitimacy from genomic studies (Whitmarsh and Jones 2010; see also Tupasela 2015). Numerous genomic studies seek to reveal the genomic characteristic of specific populations, thus claiming to provide some form of objective measure and delineation between different groups of people (cf. Reich et al. 2009). Similarly, Igo (2007) has argued that different types of population surveys, polls and gallops and other studies help construct the ways in which the population and their perceptions and opinions are projected, giving rise to reified notions of national self. Within the field of biobanking there is a large corpus of studies examining public opinions regarding biobanks and their operations (Lemke et al. 2010; Tupasela et al. 2015; Kettis-Lindblad 2007; Hoeyer 2008; Stegmayr and Asplund 2002). These studies contribute to a standardized understanding of populations from which a number of biobanks and policies draw legitimacy from in the long-term. The collection, use and distribution of human tissue samples, which include blood and diagnostic tissue samples, from which DNA can be extracted and analyzed, has become a major political preoccupation, not only in national contexts, but also at the transnational level (Gottweis 1998) in that increasingly such sample collections are not just expected to produce commercial value (Caulfield 2012), but also seen to represent a form of national capital from which research can draw from both in a scientific sense, but increasingly in a symbolic manner. In this symbolic relation, populations are increasingly seen to be the target of research (providing new treatments and medicines), but also draw on the population for their legitimacy. As such, populations have increasingly become the target of processes of bio-objectification, whereby specific populations or groups of people are ascribed various characteristics from which biobanks and policy makers draw on for legitimacy.

In a number of countries, issues surrounding the relationship between biobanks and the research population have maintained a central role in policy-making and politics as well (Epstein 2007). In the US, for example, The Centers for Disease Control and Prevention (CDC) has placed a great deal of emphasis in developing strategies for community engagement in an attempt to improve public health, as well as improve the quality and applicability of population research (Haldeman et al. 2014). Although the use of human tissue in biomedical research is not in itself new (Strong 2000), some have argued that recent biomedical research practices using tissue sample collections constitute a new object of study within biomedical research (von Versen 2000). Internationally, the formation of networks of biobanks and protocols for standards indicates a professionalization of the field itself whereby the population and people from which samples are gathered are becoming increasingly a political preoccupation. Therefore, in order to maintain the collection and research activities surrounding biobanking efficient and validated, biobankers and policy makers need to become increasingly attuned to what the public thinks. At the same time, however, this process can give rise to what Lippmann (1993) has termed the phantom public (see also Gottweis et al. 2011). In our research, however, we have noticed that many biobanking initiatives follow somewhat different definitions and practices of public engagement, and that in many cases, the public is only one target of engagement among many others. This variation can also be seen as a way of constructing the public as either an active or passive partner in the research process.

A number of national biobanking initiatives have sought to draw on a discourse of participation and involvement in an attempt to garner broader social legitimacy (Corrigan and Tutton 2006). The public shaping of science has indeed gained an important role in current social studies of the public’s role in a number of research fields, including environmental movements and patient advocacy groups (Kerr et al. 1998; see also Fuller 1999), yet it can be argued that science is also continually constructing the public as well. Along these lines, Barry (2001), 2 has suggested that the technological society brings forth a “political preoccupation with the problems technology poses, with the potential benefits it promises, and with the models of social and political order it seems to make available.” In this context, it is unsurprising that the question of engagement practices in biobanking is of political and practical interest.

The construction of populations in relation to biobanking has been seen in a number of international commercial biobanking ventures which have been developing new ways in which to collect and analyze genetic information and connect it with self-reported health information, namely through genetic self-testing. These commercial efforts can be seen as “joint social efforts” in which populations are constructed in new ways. Companies such as 23andMe, deCODEme, as well as sites like PatientsLikeMe, have sought to develop models that utilize technologies and combine them with various forms of social media through which to engage customers in their services. This form of biobanking represents new ways of constructing populations as engaged and networked. Some authors have also noted how biobanking ventures have sought new ways in which benefits can be distributed between a broader range of actors in an effort to foster trust and legitimacy (Simm, 2005). The innovations that these business models represent seek to develop new forms of engagement with research subjects, but at the same time are based on notions of the public as active in specific social ways and thus develop forms of social interaction with customers that may someday be commercially sustainable.

For public health initiatives, participation in studies is a crucial element for success and validity. During the past decades, the levels of recruitment of participants to surveys, for example, have declined dramatically (Helakorpi et al. 2011; Raisamo et al. 2011, 17), posing a serious challenge regarding the future possibilities to gather relevant scientific and medical data for large studies. From a political and sustainability perspective this can also be seen as a major challenge to legitimacy. The dramatic decline in participation levels raises important policy concerns regarding recruitment and its relation to scientific output and innovations.

It is in this context that biobanks have perhaps drawn on previous experiences of resistance and opposition movements. The organization Mannvernd, for example, was created specifically to oppose the deCode Genetics in Iceland being given monopoly rights to use Icelandic information and as a result about 10 % of Icelanders chose to opt-out of the database. In Tonga, an Australian biotech firm Autogen ran into major opposition as a result of failures to account for cultural differences and customs in Tonga. Despite having signed a contract with Tonga’s health ministry to perform research on the population, local opposition and churches in the Pacific united to oppose such ventures without extensive prior public consultation. The opposition was primarily based on the fact that the informed consent procedures did not account for the extended family system that plays a major role in Tongan society. Other oppositions included giving patent monopolies to corporations on God-created life forms (Burton 2002, 443). These examples show the ways in which biobanking activities have given rise to the politicization of populations. Despite being examples of opposition and resistance, we believe that they also highlight the way in which biobanking initiatives have sought to ameliorate such oppositions by conducting exercises in public engagement. Yet, in relation to long-term sustainability efforts, we have observed that these engagement practices are seen more as a necessary process, which much be gone through in order to maintain legitimacy.

The lessons that have emerged from such contentious events have indicated that the collection and use of biomedical tissue samples and related information should not be taken lightly. Instead, there appears to be a movement towards a more systematic and professionalized field of experts, with specific formation on biobanking, who run and operate biobanking activities, and take engagement with various stakeholders as an important function of their overall operations. Yet the ways in which engagement is operationalized and the influence this has on the way research populations are constructed varies greatly from one context to another. In the following we will explore three examples drawn from our empirical material where we think that biobanking practices are giving rise to the construction of different types of populations. We see this process to be highly political in nature and suggest that the practical day-to-day policy work and practices of governing and running biobanks are giving rise to this politicization of populations.

### Canadian provincial biobanks

Canadian discussions on ethical, legal and social issues of biobanking and aspects of public engagement have been relatively visible internationally. Prominent Canadian experts of these issues have been involved in setting up the governance of biobanks and have participated in international ethical discussions (Caufield and Knoppers 2010). Numerous surveys about public opinions in different provinces have been produced (Caulfield et al. 2012, Pullman et al. 2012) and biobanking efforts such as CARTaGENE in Quebec or BC Generations Project and BC Bio Library in British Columbia have been active in consulting stakeholders prior to the launching of the biobank and consequently constructing a certain type of population (Godard et al. 2004, O’Doherty and Burgess 2009, O’Doherty et al. 2012). In addition, Canadian medical genetics has sought to connect various populations and their historical roots to the possible future medical treatment regimes of the predominantly francofone population by suggesting that these populations have unique actionable genetic characteristics that can be used in diagnostics and treatment (Laberge et al. 2005).

Our interviews, as well as literature on public opinions in Canada, indicate that public attitudes in the different provinces have been mainly very positive towards biobanking and biomedical research. Canadians regard biobanks as public goods that contribute primarily for public benefit. This view has been manifested also in the literature where biobanks are framed as big public and local projects: “large state projects take on a civic character. They are not simply research on humans, but represent significant public investment and interests.” (O’Doherty et al. 2011).

On one hand this picture of the content and willing public has been formed through opinion surveys, on the other is based on more active engagement and interaction with the public. For example, in the beginning stages of CARTaGENE, a partnership model for the public engagement was promoted (Godard et al. 2004). In the BC generations project a considerable effort has been made to engage the public in the decision making of the biobank (O’Doherty and Burgess 2009). Despite of aims, all biobanks have not however been able to create or sustain a partnership model where the public actively engages with biobanks. Biobankers we interviewed acknowledged that they could do more with public engagement.

“There was some sort of public consultation before the beginning of the project. But they had targeted selected groups of stakeholders and experts to sort of define the roles of the project and the main objectives of the project. It wasn’t a public, it wasn’t open to the public per se.... I have to say we haven’t been really strong at working with the community.” (Interview with epidemiology director of a biobank, Canada, 2014)

The active promotion of the engagement of the public has, however, constructed a type of population that is positive, willing to contribute to common good but is not too interested, or to put it in another way, has enough trust towards the institutions involved in biobanking. This has served the purposes of the biobanks well as they have been launched and are functioning without any major resistance or public controversies.

People do not have any major concerns” (Interview with biobank manager, Canada, 2014)"

A critique singled out by our interviewees was that people are interested in getting information about their research results. Research on participants from CARTaGENE also showed that people would be motivated if they received research results (Godard et al. 2007). The big provincial biobanks such as CARTaGENE or the Tomorrow Project do not, however, by default return individual research results – only measurements done at the time of sample taking or recruitment.

Another aspect in the construction of Canadian biobank populations is that they are framed as being very nationalistic as regards to the provinces. The biobanks themselves promote the idea of provincial nationalism by stressing how the research can help the local populations (Laberge et al. 2005). CARTaGENE advertises its goals on the web pages as “for the health of Quebecers and future generations”. The Tomorrow project from Alberta talks about how the project can help in treating cancer: “cancer affects everyone in some way. Here in Alberta, 1 in 2 Albertans will be diagnosed with cancer in their lifetime, and 1 in 4 will die from it.” Biobankers we interviewed recognized that people were motivated by the possibility to contribute to the public good or health in their own province. Similar tendencies can also be seen in relation to Finnish biobanks (Tupasela and Snell 2012).

During recent years there have been major transformations occurring in the biobank sector, both internationally as well as inside Canada. First of all, the locality of biobanks is becoming increasingly blurred as the biobanks are more actively and openly becoming parts of networks, such as the Canadian Partnership for Tomorrow Project (CPTP) which is a multimillion dollar research platform with currently almost 300 000 participants from different parts of Canada. The core of the platform is comprised of five regional biobanks: CARTaGENE (Quebec), Ontario Health Study (Ontario), Tomorrow Project (Alberta), Atlantic Path (Atlantic provinces), and BG Generations Project (British Columbia). Biobankers we interviewed expressed that this has caused discontent in some participants as the focus of the project has shifted from provincial to federal. A notable part of those recruited earlier did not want to re-enroll in the biobank as it was becoming part of a federal network.

“They joined in an Albertan project. Alberta likes to be independent from the federal government. It is a challenge for being a part of a federal project.” (Interview with biobank manager, Canada, 2014)

A second change is related to the increasing pressure to return individual research results. It is not only a motivational factor coming from the public but also a vivid topic of scientific, ethical and legal discussion internationally and in Canada. This has created pressure to change the perception of the ideal populations from a content, nationalistic population that is motivated by public good to one that is more interested in the individual research results and has to be more internationally open-minded. From a policy perspective this has become a challenge since there is a lack of international normative standards on the return of individual research results (IRR) and incidental findings (IF) (Zawati and Knoppers 2012).

In summary, the construction of the population by Canadian biobanks has been characterized by an initial depiction of the public as content and participative. This, with the evolvement of the several national and regional projects, has developed into a different scenario. Firstly, donors and patients have grown to be less content discontent with regional projects as they became part of national federalized biobank network associations. Secondly, the lack of direct and personalized results has also affected their attitude towards the projects. These changes have result into a lowering of the willingness in the participants to re-enroll for future biobank projects.

### The public as a secondary engagement target: constructing populations by default in the Spanish context

In our interviews with Spanish biobankers, engaging the general population, patients and donors often seemed to be an activity relegated to the background by other priorities. While most biobank literature pays attention to the engagement with the public and, which we also sought to explore in our interviews, the conversation always turned towards other actors that also need to be engaged, but who are usually not considered in terms of engagement (Cañada, Jose A., Aaro Tupasela, and Karoliina Snell: Beyond and within public engagement–Communities biobanks engage with, submitted). Some of these communities were other biobanks, industry, doctors, clinicians, researchers, funding organizations and public administration. Therefore, the general population is constructed almost by default in the Spanish context in relation to other more prominent actors. However, the focus on other engaged communities, has had positive consequences as the “Spanish design” of biobank networking, as pointed out by one of our interviewees, has often served as an example for the international community (see also Romeo Casabona et al. 2011).

Regarding public engagement, in our interviews with Spanish biobankers, it became apparent that in some of the regions, framing biobanks in terms of population-based collections seemed to be a sensitive issue:

It is a very sensitive topic, you always need to be very careful, nowadays it is less complicated, but there is always that dogma about mixing science and politics. We set it out very clearly as a research project, which requires the collection of a series of very concrete samples […], we try to unlink science and politics. (Interview with biobank manager, Spain, 2014)

In other countries, such as the cases of UK Biobank in the United Kingdom, deCODE in Iceland, or CARTaGENE in Quebec, claiming to aim at representing a region’s or nation’s genome may appear much more legitimate, and even useful for the sake of increasing funding and donors. This situation seems to be different in cases of countries with a complicated nationalistic past, as it is, for example, the case of Germany (Schneider 2008), despite the fact that a biobank framed as population-based gives increased scientific appeal and power to sample collections. The history of nationalistic disputes that characterizes the political atmosphere in Spain may have not allowed for the construction of populations as one unitary whole. Biobanks need to, therefore, define themselves and their competitive advantage through other means and interactions that do not include populations: economic interactions with industry, associative interactions with other biobanks or researchers become imperative in defining the biobank’ identity. At the same time, in Spain, the nationalistic conflicts are often dealt with caution in research so as to not politicize such research.

On the other hand, from a utilitarian perspective, to engage directly with patients and donors is not the optimal course of action in the Spanish biobanking scene. Recruitment is usually done through third parties, such as clinicians. This allows biobanks to distance themselves from possible privacy controversies. As one biobank manager noted in one of our interviews:

Most of the researchers are against this law [the Biomedical Research Law]. Because they think that it hinders research, because before, researchers would manage the samples themselves and this law is, especially, protecting the rights of donors and patients. And there have been quite many protests from the research community. […]. [The Biomedical Research Law] does not benefit [the donor], it protects [her or him]. It does not benefit [her or him] because the donor does not perceive it, but it protects, because it is a law that establishes a very extensive informed consent document. I believe that in some way they cover their backs in front of a still uncertain future regarding mass sequencing and genic therapy and such (Interview with biobank manager, Spain, 2014)

In this interview excerpt we can see how the constructed population is different depending on who is constructing it, biobankers or policymakers. Biobanks seem to see donors as something they need to reach; something they depend on, but that brings hindrance to research. In terms of policymaking they are constructed as something to protect, but also as a threat. If the law does not cover all sorts of uncertain facts related to biotechnology and research, it may backfire in the judicial system. The lack of public controversies in Spain, if we compare with the cases of UK or Iceland where there has been lively debates concerning large national biobanking initiatives, could be one of the reasons of why biobanks do not see interacting directly with the public as a priority.

In our interviews, privacy concerns did not appear as a real possibility and, in some cases, samples were not anonymized, but just coded, as data was not considered to be sensitive. This has probably allowed biobanks to focus on other types of engagement rather than public. It is easy to see in our interviews certain precaution with regard to their interaction with patients. This influences heavily the way biobanks engage with patients and donors, often preferring to leave the direct contact to other actors, such as clinicians, or to rely on press. As it was noted in one interview:

We have not done that sort of relationship exercise [with society]. What we actually do, every time we can, is to appear in the media to divulgate the idea of the biobank. (Interview with biobank manager, Spain, 2014)

But even this indirect engagement is something that is usually placed in the to-do list of the biobanks. The importance of patients and donors is recognized in discourse as they are biobanks’ “first bulwark”, but public engagement is not so easily achieved. All of our interviewees saw the objective of engaging with society as a pending objective describing one of them their relationship with society as “little or null” and being “one of their pending objectives” as “most of people do not know what biobanks are”.

Nevertheless, the secondary role played by patients is not necessarily an essential feature of the Spanish biobanking scene, but a consequence of complicated socioeconomic entanglements resulting from recent Spanish history. Besides nationalistic disputes, the economic crisis has provoked the current moment to not be the best for research in Spain, as noted by one of our interviewees. This forces biobanks to engage with other actors such as other biobanks, the international community or the industry. In one interview, it was noted that

There’s a clear survival challenge, to depend on public funding with the current economic situation in Spain is very complicated and one of our main concerns is to penetrate the reproductive industrial fabric, so to be able to give steps so this platform, in case the official funding would cease, could go on being viable and sustainable over time (Interview with biobank network coordinator, Spain, 2014)

The types of population constructed in our interviews with Spanish biobankers are the result of two processes. On the one hand, the sensitiveness of creating population-based biobanks and, on the other hand, the necessity of prioritizing other types of engagements for the sake of sustainability, leaving the general population on a secondary role for biobanks and leaving their engagement to third actors. Therefore, rather than conceptualizing an “ideal type of population”, Spanish biobanks seem to conceptualize them by default. Their absence, influenced by the circumstances, is what defines them mostly as providers of biological samples rather than as social actors and, therefore, stakeholders of the biobanking scene.

### Compliance and Consensus in Finland

In Finland, which is a relative smaller country than Canada and Spain, the construction of the public has been the result of a strong medical profession that has drawn on the authority of the welfare state. Finland has been conducting research into national health risk factors since the 1950s and collecting DNA-samples for such purposes since the beginning of 1980s (Aromaa et al. 2002, 7). According to a study funded by the Finnish National Technology Agency Tekes, Finland has over 190 000 samples within ten of its most significant epidemiological cohort studies (Technomedicum 2004). This represents about 3.6 % of the country’s population, which in comparison to the UK Biobank’s 500 000 samples from a population of over 60 million represents only under 1 % sample of the population. According to the report, these samples and the related health information could be used far more efficiently in the study of the human genome, diseases, as well in the development of pharmaceuticals and treatment. The report also sees genome research as uniting science and industry in a way that will give Finland an edge over similar competing projects elsewhere in the world. In addition to the epidemiological cohort collections, Finland has pathology collections that amount to well over 2 million samples.

Whereas the authority of the medical profession has been under attack in some countries and contexts (Hess 2004; Brown and Zavestoski 2004), the Finnish medical profession has always maintained a very strong presence in the way policy has been developed in the country (cf. Tupasela 2007). In part, this trajectory is similar to many of the other Nordic countries where the welfare state has played an important role in improving the quality of people’s lives. Unlike Iceland, however, where the setting up of deCode and the national legislation that would subsequently support it, there has not been any major controversies in Finland regarding biomedical policy. In part this can be explained by the lack of a larger national biobank project, but also in part by the strong medical profession which has traditionally sought to construct a very willing and eager population. Like some other countries, Finnish biomedical research has drawn on rhetoric of common historic and thus genetic ancestry whereby Finnish genes are seen to form an important part of developing drugs and treatments for Finns (Tupasela 2015).

In many of the policy documents that we examined the population was constructed in a very positive and compliant way. In a 2003 policy document, for example the general attitudes of various stakeholders were described in the following way.

Compared to many other countries or regions, one of the Nordic countries’ greatest strengths is its extremely wide-ranging and high-quality population-based registers, and patient and sample databases, whose compilation has been extremely well-received by decision-makers, researchers and the general population. (Academy of Finland 2003, 16)

Although the Nordic countries, in general, have always sought to draw attention to the excellence of their medical records and registers, Finland has also sought to integrate the discussions related to use and management of tissue collections in relation to their role in the national innovation system. In this sense, the public is ascribed a willing and positive attitude in relation to the commercial opportunities that relate to biobanking. Like other Nordic countries Finland has also conducted a number of studies into public perceptions of biobanking and tissue use (Tupasela and Snell 2012; Hemminki et al. 2009; Sihvo et al. 2007). In many ways, these studies have bolstered the notion of a unified perception of biobankers despite the fact that there are differences in public opinion towards the biomedical use of tissue collections.

In a number of our interviews, with both policy makers and researchers, it was noted that the use of tissue collections was seen as a potential source for commercial opportunities. As one publication related to biobanking noted.

As a counter question one can ask whether it is justified from the perspective of Finnish taxpayers not to exploit the enormous commercial potential which Finnish biomedical research has produced during the past years? (Käpyaho et al. 2004, 10)

This type of argument format was quite usual in the Finnish documents where, the various ethical and legal issues were discussed on their merits and relevance in relation to public expenditure, as well as commercial potential (cf. Tupasela 2008). Much like the Spanish biobanks, engagement with the public was not seen as the primary task within biobanking activities, but unlike Spain, however, Finnish policy makers and researchers did not appear to need to distance themselves from the public to such a great degree. Instead, in many of the documents we looked at, as well as in the interviews, the tone was very direct in relation to presenting the function of public funding in relation to biobanks.

Research sample collections collected with public funding, diagnostic sample collections and related information can be seen as being a part of the infrastructure that supports research and innovations, whose efficient utilization can be seen to benefit the whole society. […] In biobanking research, the interests of the researcher, the research participant and society are parallel. Biobank research produces significant new research findings. The translation of these findings into products and services that contribute to public health requires also partnerships with the private sector. Finland’s prosperity is based on the generation of innovations, their up-take and the creation of new businesses. (Sosiaali- ja terveysministeriö 2007, 13)

The last couple of years have, however, created a new situation for biobank research in Finland. A new Biobank Act (Act 688/2012) came to force in September 2013. The act is one of its kind in Europe as it regulates directly and only biobanks of all types–from population to clinical and disease based biobanks and public as well as private biobanks. The Biobank Act defines criteria for biobanking and its passing has resulted in a situation where all research infrastructures that called themselves biobanks prior to the enactment of the Biobank Act have to apply for permit to function as a biobank as defined by the act. This has created lots of activities in the field and most old tissue collections will be or have already started the process of transferring to biobanks.

The two first biobanks–as defined in the act–got their permissions to start operation in early 2014 from the National Supervisory Authority for Health and Welfare that supervises biobanks. The two new biobanks are Auria Biobank (a clinical biobank in the Turku hospital district) and THL Biobank (a national population-based biobank, to which it transfers its major longitudinal cohorts). Auria Biobank’s goal is to get new samples from every patient enrolled in the hospital. During the retrieval of a diagnostic sample a surplus sample will be given to the biobank after receiving consent from the patient. In addition, it is transferring old sample collections of the hospitals, as well as research project samples to the biobank. Unlike Iceland or the UK where the setting up of large biobanks have spurred major public and professional debates, the Finnish example has proven quite the contrary where there has been very little or no public discussion related to the transfer of diagnostic samples or legacy collections into the new biobanking framework. One could compare the move akin to taking all the samples in the London area and transferring them to UK Biobank, for example, without any public debate over the move. According to the Auria Biobank website.

In biobank research the interests of the researchers, the research subject and society are parallel. Finnish welfare is based on the development of innovations, their uptake, as well as the development of new businesses (Auria Biobank 2014)

The idea that in biobank research the various stakeholders hold the same values and goals is somewhat problematic, especially given the large amount of evidence from around the world regarding differing perspectives, yet the statement is emblematic of the ways in which biobankers in Finland tend to view their role in society, as well as their relationship with various stakeholders, especially research subjects and the public, in particular.

This lack of public debate reflects the nature of the public engagement and the power of the medical community in constructing policy. It also reflects the ways in which the public have been constructed in a compliant and supportive manner, whereby policy discussions are from the outset framed in a way that is difficult to problematize.

## Discussion

In our research of biobank engagement strategies in six countries we have made a note of the different ways in which various biobanks, as well as biobanking policies, have sought to construct or bio-objectify the populations from which they source their samples. We have observed that in much the same way that the people from whom the samples are sourced help to form the identity and scope of the biobank itself, so does the biobank and the policies and governance environment in which they operate help to construct the population itself. We see these processes of bio-objectification as attempts at stabilizing the populations from which they derive both their samples and legitimacy from. We also see this iterative process between historical, political and scientific narratives to be highly dynamic and contextualized, giving rise to different types and forms of bio-objectification, which are generated though varying styles and forms of engagement strategies.

The three countries from which we draw our examples – Canada, Spain and Finland – are very different in terms of the historical and political trajectories in which their biobanking activities have operated. The Canadian federalist system has emphasized the regional character of biobanking drawing on the specificities of each regions population to highlight the significance that biobanking has for each regions population. As the various biobanks have sought to construct a very positive picture of the various populations, it is only very recently, however, that these policies have come under challenge as regional biobanks are integrated into federal and international networks and people are expecting to get more personal results back from the research system.

In Spain, recent political and economic challenges have prompted a very different approach to engagement and thus contributed to the construction of a very different type of public, which is far more distant and passive in relation to biobanking activities. Biobanks have often explicitly sought to keep the public at a distance and instead preferred to develop stronger links with other actors, such as physicians and hospitals who act as buffers for engaging with the public, as well as the people from whom samples are collected. Although public engagement is described as useful and necessary, it is far from being a top priority for Spanish biobanks and, given the scarce resources biobanks count on, it seems like direct engagement strategies will not be carried out soon.

In Finland, the construction of a positive and willing population, whose interests are parallel to those of researchers and society reflects a strongly medically driven and controlled image of public perceptions. Much like in Spain, where there has been very little public discussions surrounding biobanking policies, the Finnish strategy has sought to develop a very expert led agenda concerning public interests. Finland and Spain differ, however, in the sense that Spain has not undertaken many efforts to gage public perceptions through surveys, for example, while Canada and Finland are more similar in this sense. As a result, recent Canadian and Finnish policy discourse tends to draw on its legitimacy from such studies, which would also suggest that biobanking efforts also draw on other sources through which the public is constructed, such as in the case of Spain.

Bio-objectification has been seen as a way of stabilizing conceptions of patients within medical research (Douglas 2012). Our study suggests that the forms of stabilization of populations in relation to engagement practices are regionally and locally contingent, whereby historical, political and scientific specificities can give rise to different ways in which populations are constructed and engagement conceptualized. Igo’s notion of a mass society constructed through surveys and questionnaires is informative in many ways with regard to the construction of populations from which biobanks draw from, yet limited, in that not all countries, regions or institutions seek to draw on surveys for further legitimacy. Instead, biobanks draw on a diverse range of material from which they seek to draw legitimacy from. What is significant about these processes, however, is that they seem to be very static. This creates a number of problems including an inability to quickly react and adapt to changes in public perceptions, as well as the creation of particular types of worldviews within specific contexts.

We believe that our cases show that the construction of populations through the collection of biological samples does not only take place through the collection and research process, but also through the interaction with such populations. This means that biobanks, besides defining populations by analyzing the biological characteristics of a population or by collecting population data, also describe those populations through the way they interact with them. Similarly, the development of biobanks draws heavily on the historical and political context in which they have developed. Bio-objectification cannot, therefore, be seen as a unidirectional process where science acts upon populations, but rather an iterative process where identity and legitimacy are co-constructed. The local nature of the bio-objectification process may pose a problem in relation to attempts to standardize and stabilized ethical, legal and social practices surrounding biobanks internationally. Such attempts may not take into account sufficiently regional differences and context, but rather suppose a one-size-fits-all solution.
